# Long Term Performance of a Bi-Directional Neural Interface for Deep Brain Stimulation and Recording

**DOI:** 10.3389/fnhum.2022.916627

**Published:** 2022-06-09

**Authors:** Scott R. Stanslaski, Michelle A. Case, Jonathon E. Giftakis, Robert S. Raike, Paul H. Stypulkowski

**Affiliations:** Medtronic, Minneapolis, MN, United States

**Keywords:** deep brain stimulation, local field potentials, evoked potentials, deep brain sensing, long term brain signal stability

## Abstract

**Background:** In prior reports, we described the design and initial performance of a fully implantable, bi-directional neural interface system for use in deep brain and other neurostimulation applications. Here we provide an update on the chronic, long-term neural sensing performance of the system using traditional 4-contact leads and extend those results to include directional 8-contact leads.

**Methods:** Seven ovine subjects were implanted with deep brain stimulation (DBS) leads at different nodes within the Circuit of Papez: four with unilateral leads in the anterior nucleus of the thalamus and hippocampus; two with bilateral fornix leads, and one with bilateral hippocampal leads. The leads were connected to either an Activa PC+S^®^ (Medtronic) or Percept PC^°ledR^ (Medtronic) deep brain stimulation and recording device. Spontaneous local field potentials (LFPs), evoked potentials (EPs), LFP response to stimulation, and electrode impedances were monitored chronically for periods of up to five years in these subjects.

**Results:** The morphology, amplitude, and latencies of chronic hippocampal EPs evoked by thalamic stimulation remained stable over the duration of the study. Similarly, LFPs showed consistent spectral peaks with expected variation in absolute magnitude dependent upon behavioral state and other factors, but no systematic degradation of signal quality over time. Electrode impedances remained within expected ranges with little variation following an initial stabilization period. Coupled neural activity between the two nodes within the Papez circuit could be observed in synchronized recordings up to 5 years post-implant. The magnitude of passive LFP power recorded from directional electrode segments was indicative of the contacts that produced the greatest stimulation-induced changes in LFP power within the Papez network.

**Conclusion:** The implanted device performed as designed, providing the ability to chronically stimulate and record neural activity within this network for up to 5 years of follow-up.

## Introduction

Deep brain stimulation (DBS) continues to evolve toward the standard of care for medically refractory movement disorders including Parkinson’s disease, essential tremor, and dystonia (Miocinovic et al., 2013) and is in the early stages of adoption in the treatment of pharmaco-resistant epilepsy (Laxpati et al., 2014). Clinical investigations of DBS to treat other neurologic and psychiatric disorders also continue, despite recent setbacks in larger, industry-sponsored clinical trials (Dougherty et al., 2015; Holtzheimer et al., 2017). Although initially approved in the late 1990s, the delivery of DBS for movement disorders today remains virtually unchanged from the early reports of Benabid and colleagues (Benabid et al., 2009). Technology improvements have included more robust hardware, rechargeable batteries, and recent innovations in electrode design (Steigerwald et al., 2016), however, the fundamental therapy remains a tonic, continuous delivery of a high-frequency pulse train, of fixed amplitude, to the targeted neural network. And unlike implantable devices used for cardiac therapies, which can measure and report the physiologic effects of stimulation on the target organ, commercial DBS systems for movement disorders remain “open-loop,” relying solely on patient and clinician feedback for parameter adjustment.

The concept of closed-loop or adaptive DBS (aDBS), using local field potentials (LFPs) from basal ganglia nuclei as a control signal, was described over a decade ago (Rossi et al., 2007; Marceglia et al., 2007). Subsequently, aDBS has been investigated in pilot acute, or semi-chronic studies in Parkinson’s patients (Little et al., 2013, 2016; Priori et al., 2013) using percutaneous access to implanted DBS leads. However, the broad translational potential of aDBS is still debated, particularly with respect to the chronic accessibility of sufficient LFP control signals. We previously reported on the design (Stanslaski et al., 2009, 2012), and initial chronic performance in animals (Stypulkowski et al., 2013) of a fully implantable stimulation and recording system (Activa PC+S^®^) that permits sampling of brain electrical activity, and also demonstrated its capability to deliver closed-loop DBS using this LFP based approach (Afshar et al., 2013; Stypulkowski et al., 2014). Here we provide an update on the chronic long-term (up to 5 years) performance of this system in the initial ovine subject cohort, and report on a second cohort implanted with directional DBS leads and Percept PC^®^.

## Materials and Methods

This study was conducted at the Physiological Research Laboratory (Medtronic, Inc; Minneapolis, MN) under a protocol approved by the institutional Animal Care and Use Committee. Detailed implant and stimulation/recording methods have been previously reported (Stypulkowski et al., 2011) and are briefly summarized here.

**Figure 1:**
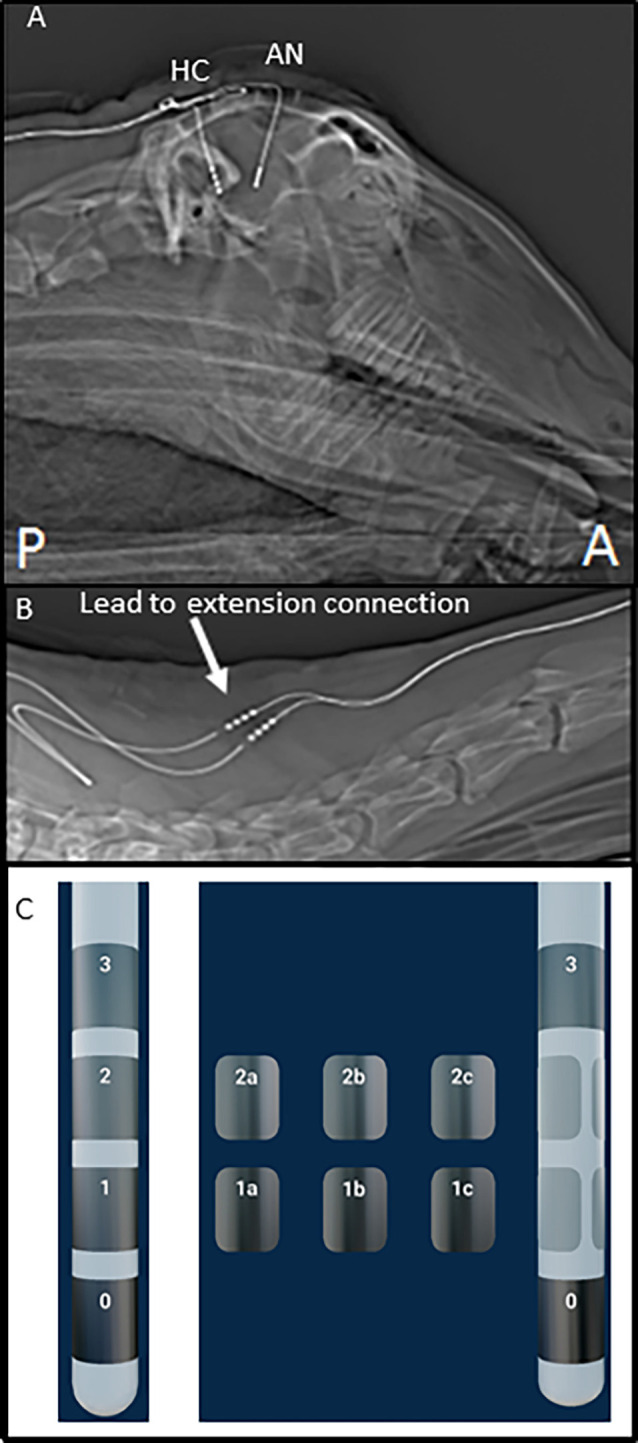
Post-operative images of deep brain stimulation (DBS) implant. **(A)** Top panel shows the sagittal X-ray image of the head of one subject with DBS leads in place. **(B)** This panel illustrates the position of lead-extension connector (arrow) at the base of the neck, near the junction of cervical, and the thoracic spine. **(C)** This panel illustrates the two lead types used in this testing and electrode numbering.

The initial cohort of three adult Polypay mixed breed sheep was implanted with unilateral DBS leads in the anterior nucleus of the thalamus (AN; Model 3389) and hippocampus (HC; Model 3387) using MRI-based and frameless stereotactic methods similar to those used in human surgery (Holloway et al., 2005). Subsequently, four additional animals were implanted with investigational directional DBS leads (1-3-3-1 configuration): two with bilateral fornix (FX) leads; one with bilateral HC leads; and one with unilateral AN and HC leads. The leads were connected to DBS extensions (Figure 1) which were tunneled to a post-scapular pocket and connected to either the investigational Activa PC+S^®^ (Medtronic) neurostimulator/recording device (initial cohort) or Percept PC^®^ (Medtronic) device (second cohort). Post-operative images were collected to confirm the stereotactic location of the DBS leads. The electrode configuration for the 4-contact 3389 and 3387 leads are labeled E0, E1, E2, and E3 and are shown in Figure 1C. While the directional 1-3-3-1 DBS leads are labeled E0, E1abc, E2abc, E3 and are shown in Figure 1C.

### Stimulation and Recording

The implanted system allowed for stimulation and recording from both leads, with specific contact configurations selected *via* programmable telemetry interfaces. The implanted system always records differentially between two electrodes for example E0-E3. Recording parameters (sampling rate, filter cutoffs, center frequency, bandwidth, etc.) for time domain signals were set *via* a custom-designed programming interface tablet. Stimulation parameters were controlled using a standard DBS Physician Programmer (Model 8840; first cohort) and a custom-designed programming interface for the directional leads in the second group.

In-person recording sessions with stimulation in the initial cohort were conducted for approximately 2-h periods with the animals awake and resting in a sling. Hippocampal evoked potentials (EPs) were elicited by trains of stimuli delivered to the thalamic lead (5 Hz, 30 s duration, 1–7 V, 120 μs pulse width). The device also permitted simultaneous LFP recordings from the two leads to be triggered at pre-set times, when the subjects were in their free-roaming, home environment. All time domain signals were recorded by the implanted device (200–800 Hz sampling rate; 0.5 Hz HP, 100 Hz LP filters), downloaded, and analyzed offline. At the time of this report, chronic implant durations for all three subjects ranged from 6 to 7 years.

Similar LFP recordings were obtained in person in a sling (with stimulation) and remote free-roaming conditions in the second group. Remote recording capability was a new capability added to enable remote data collection from home. The 8-contact directional leads in these animals allowed recording from a selected montage of 15 bipolar pairs. To assess the effects of directional stimulation on LFP activity, monopolar DBS (1 mA, 300 μs, 100 Hz, 10 s duration) was applied to each segmented contact in a randomized order. Bipolar sensing was configured either around the stimulation electrode or with angular contact pairs (e.g., E1a-E1b, etc.). Baseline LFP data were recorded for 20 s before stimulation and up to 10 min after stimulation.

### Data Analyses

Electrophysiologic data was analyzed using Acqknowledge 4.1 software (BioPac Systems). EPs were averaged offline using the stimulus artifact as the trigger, as no external time sync signal was available. In some cases, within-subject records were aligned approximately to the main peak in the EP due to variability over time in the stimulus artifact used as the averaging trigger. LFP spectrograms (intensity of instantaneous frequency vs. time; Hann window) were generated with Sigview software (v3.1.1) and are displayed using a logarithmic Z-axis with color representing relative intensity. The spectral density of the LFP signals was calculated with MATLAB R2017a (intensity of instantaneous amplitude vs. frequency; Hann window) and is displayed with a logarithmic Y-axis in units of μV/√Hz vs. frequency. The LFP power was measured from each contact segment on the directional leads, ranked according to magnitude, and plotted over time. Additionally, the response to stimulation was examined by comparing median LFP power (avg/s.d.) at baseline (pre-stimulus) and each minute after stimulation for each segmented electrode.

## Results

### Stimulation Evoked Responses

**Figure 2:**
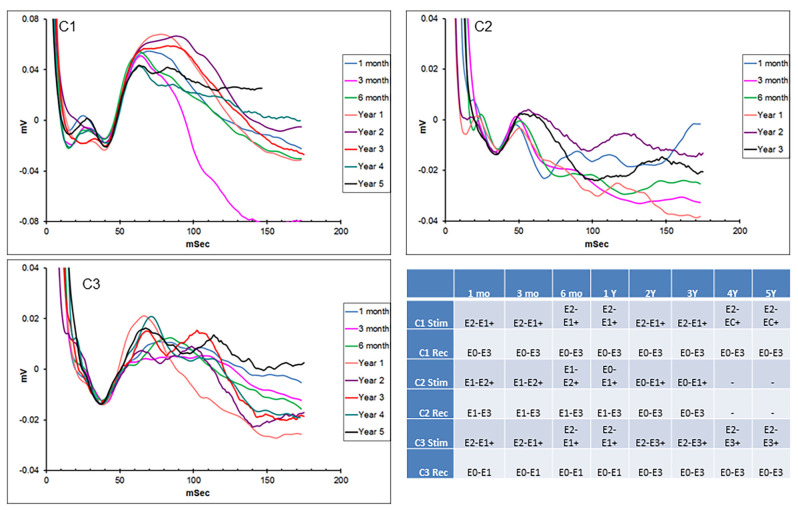
Chronic hippocampal evoked responses. Evoked potentials (EPs) recorded at multiple time points from the three subjects in response to anterior nucleus (AN) stimulation. Slight changes in electrode contact pairs were made in some cases due to lead breakage or possible electrode shift over time. Subject C1: recording (HC E0-E3), stimulation (AN E2-E1+, 5 V; AN E2-C+, 1.5 V); subject C2: recording (HC E1-E3/E0-E3), stimulation (AN E1-E2+; E0-E1+, 5 V); subject C3: recording (HC E0-E1/E0-E3), stimulation (AN E2-E1+, E2-E3+, 5 V). HC, hippocampus.

Hippocampal EPs in response to thalamic stimulation were recorded for up to 5 years in two of the initial subjects and 3 years in the other, due to lead breakage. Figure 2 shows representative EPs from each animal at different time points over the duration of the study. As described in earlier reports, there were subtle differences in the morphology and main peak latency (35–40 ms) of the EPs between subjects. These across-subject variations were expected, due to slight differences in hippocampal lead location and recording contact configuration relative to the source dipole of the EP. In addition, the required use of stimulus artifact as the averaging trigger also resulted in several milliseconds of difference in the absolute latencies measured. Within-subject recordings, however, were consistent throughout the duration of the study period with respect to morphology, amplitude, and latency. In some cases, changes in stimulation or recording contact configurations were necessary due to lead breakage (subjects C1—year 3; C2—years 1 and 3) or presumed lead shift (subject C3) over time (details in the figure legend). In addition, the morphology of the evoked response changed in subject C1 at 3 months. This is thought to be due to minor threshold shifts over time and not due to a lead breakage. Despite these minor modifications, in all cases, it was possible to record a consistent, reliable hippocampal EP with AN stimulation, for an extended period of years in these subjects using the implanted device.

**Figure 3:**
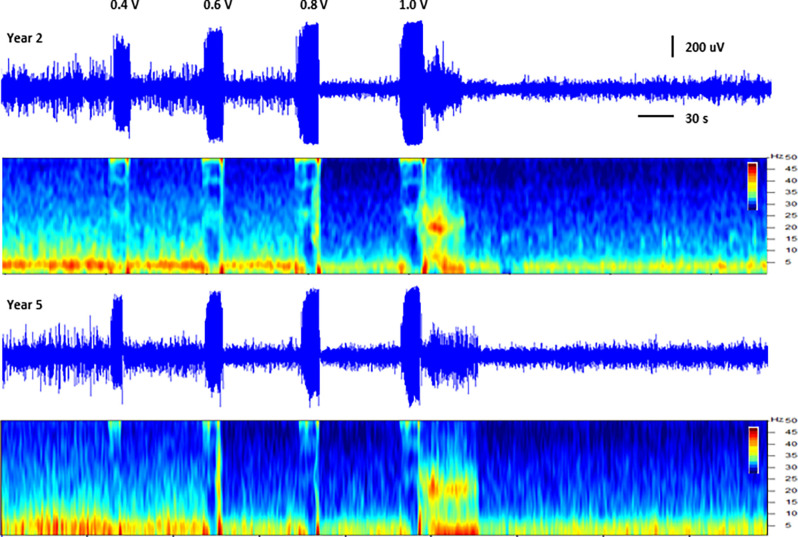
Direct hippocampal stimulation. Hippocampal local field potentials (LFPs) and spectrograms (40 dB scale) recorded at approximately 15 months (year 2, top panels) and 57 months (year 5, bottom panels) post-implant from subject C1 illustrating the response to stimulation of the hippocampus. Stimulation was delivered to contacts HC E1-C+ at the amplitudes shown (50 Hz, 10 s ramped burst, 300 μs PW) while recording from contacts HC E0-E2. The large signal below the amplitude labels represents the recorded stimulus artifact.

In addition to thalamic stimulation, the response to direct hippocampal DBS was also evaluated in these subjects over the course of the study. Hippocampal LFPs recorded from one subject at two different time points separated by approximately 3.5 years are shown in Figure 3. Each trace shows a series of increasing amplitude stimulus bursts delivered to the hippocampus and the resultant effects on LFP activity. At the lowest amplitude (0.4 V) there was little to no apparent effect; as the stimulation amplitudes were increased (0.6 and 0.8 V), the inhibitory threshold was reached and suppression of ongoing activity following each stimulus burst was observed. As the amplitude was further increased, the after discharge threshold (1 V) was exceeded and a large excitatory burst of activity occurred. The thresholds for these inhibitory and excitatory effects produced by direct hippocampal DBS were remarkably stable in this subject over this extended period of time.

### Spontaneous Local Field Potentials

**Figure 4:**
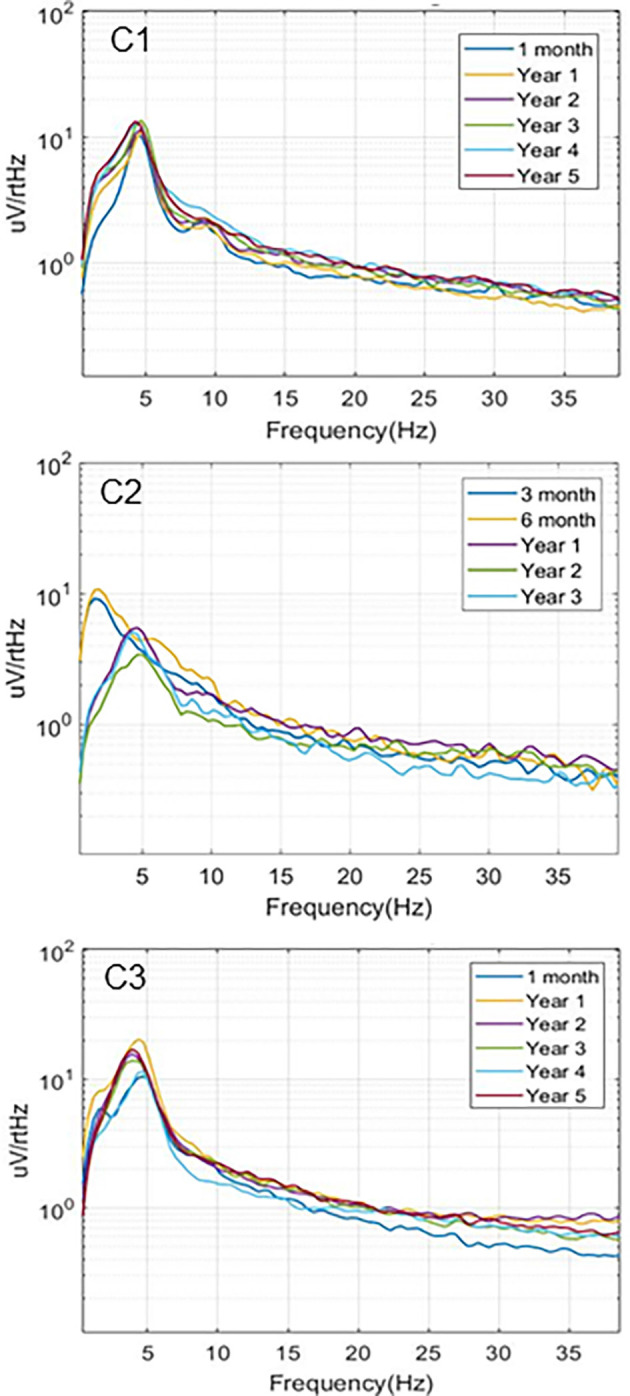
Local field potentials. Representative examples of hippocampal LPF power spectra recorded over the study period for the three subjects. C1 (HC E0-E3) and C3 (HC E0-E3) recordings exhibited predominant theta activity. Initial recordings from C2 (HC E1-E3) contained mainly sharp wave activity while later recordings from a different electrode pair (HC E0-E3), due to lead breakage, were more theta dominant.

Simultaneous recordings of spontaneous local field potentials from the thalamus and hippocampus were also collected at regular intervals in the initial subjects. These timed recordings (typically 5 min duration) were set to trigger throughout the day when the subjects were in their home environment, and, therefore, included a variety of behavioral states, ranging from sleep to fully active. Figure 4 illustrates the hippocampal power spectra for example recordings from the three subjects at various time points in the study. Each recording represents an averaged power spectral density plot for 30 s of data. The records selected represented the most common type of neural activity pattern observed for each subject in these sampled recordings, which could vary considerably based on behavioral state, time of day, etc. Two of the subjects (C1, C3) exhibited predominant hippocampal theta activity, with a strong spectral peak at 4–5 Hz. Initially, the recordings from subject C2 (contacts HC E1-E3) contained primarily hippocampal sharp wave activity, with a dominant lower frequency peak in the power spectrum. A lead breakage (contact HC E1) occurred in this subject at approximately 14 months, which required a shift to recording pair HC E0-E3. With this recording configuration, the spectral power shifted to a much more theta dominant profile, similar to that observed in the other two subjects. Just after year 3 in this subject, the remaining contacts on the hippocampal lead became non-functional, and further recordings were not possible.

**Figure 5:**
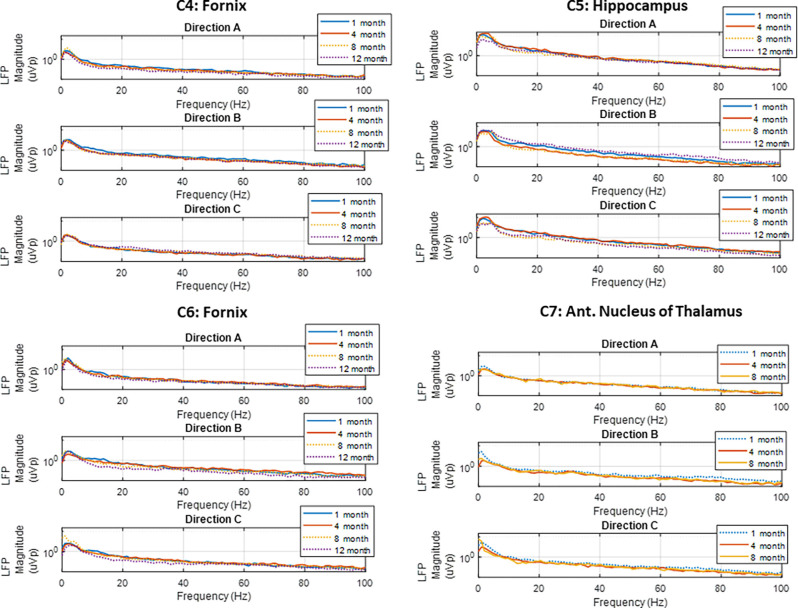
Local field potentials. Representative examples of LFP power spectra from directional pairs (E1a-E2a, E1b-E2b, E1c-E2c) recorded over the study period for the four subjects implanted with directional leads. Recordings showed predominant theta activity in all nodes monitored. The dotted line denotes the free-roaming condition, and the solid line denotes the sling condition. Each line represents one 30 s sense recording for the representative study period.

Spontaneous LFP activity was recorded periodically from the FX, HC, and AN in the subjects implanted with directional leads, during both sling and free-roaming sessions. The recordings took place over a 2-min period, where the 15 bipolar pairs were split into three groups and each group was recorded for a 40 s duration. The corresponding segment pairs (E1a-E2a, E1b-E2b, E1c-E2c) are plotted to show the LFP activity in each radial direction (Figure 5). Each neural target in the network showed predominant theta activity with a strong spectral peak at 4–5 Hz, similar to what was observed in the subjects in the first cohort. The dominant direction of strongest LFP power tended to be consistent for similar conditions (sling vs. remote) throughout the monitoring period, up to 1 year. Two subjects with directional leads experienced a lead breakage: one in the bilateral FX subject and one in the AN-HC subject.

### Directional Stimulation

**Figure 6:**
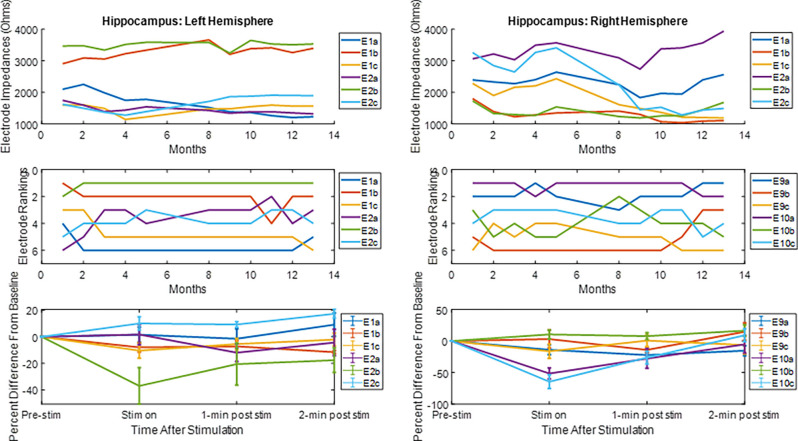
Electrode impedance and rankings and local LFP response to hippocampal stimulation. For the electrode rankings, in-person monitors took place between months 1 and 4 and remote monitors took place between months 5 and 13. For the LFP response to stimulation, the LFP response compared to baseline is plotted for the duration of the recordings. The average response and standard error are displayed. For both hemispheres, the top-ranked electrode corresponded with an electrode that had an observable decrease in LFP activity after stimulation.

**Figure 7:**
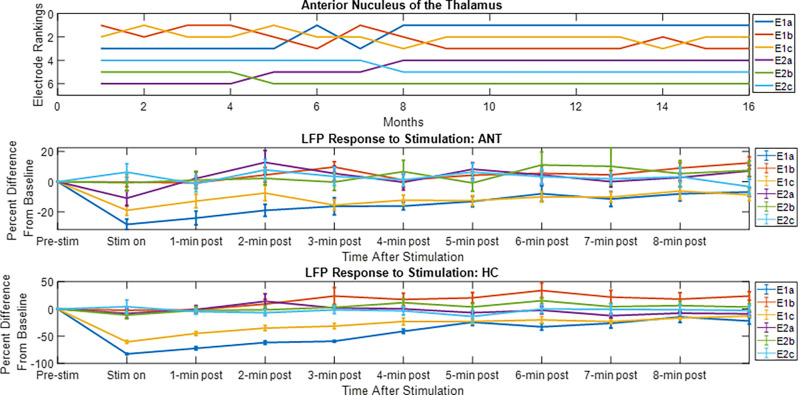
Electrode rankings and the LFP response to thalamic stimulation. For the electrode rankings, in-person monitors took place between weeks 9 and 16 and remote monitors took place between weeks 1 and 9. For the LFP response to stimulation, the LFP response compared to baseline is plotted for the duration of the recordings. The average response and standard error are displayed. For the local response in the AN and for the network response in the HC, the top-ranked electrodes corresponded with the electrodes that had an observable decrease in LFP activity after stimulation.

The LFP power-based rank over time for each contact segment in the bilateral HC subject was calculated and compared to the stimulation results (Figure 6). The top-ranked electrode for the majority of the monitoring period (E2b) in the left hemisphere corresponded with the electrode that had the greatest LFP suppression in response to stimulation. A similar pattern was observed on the right side, with higher-ranked contacts (e.g., E10a, E10c) producing greater levels of local LFP suppression. Impedance data were also collected and presented over time and shown to be in normal ranges. More on impedance measurements will be presented in the “Discussion” Section. The goal of this work was to demonstrate signal stability over time. However, to motivate future work, the coefficient of determination was calculated between the ranking of LFP power at the 4-month time point and the ranking of LFP suppression for each hemisphere, where the left was *R*^2^ = 0.1 and the right was *R*^2^ = 0.4. Figure 7 shows the same type of analysis conducted for the AN-HC subject, with stimulation applied remotely to the AN target and the LFP suppression assessed in the HC. Here again, contact segments that were ranked higher based upon spontaneous LFP power (e.g., E1a, E1c) tended to produce the greatest LFP suppression, as measured both within the Papez network (HC) and locally (AN). The coefficient of determination was *R*^2^ = 0.8 for the AN and *R*^2^ = 0.4 for the HC using the 10-month data. The results from these two subjects suggest that both the local and network response to stimulation tended to align with the rankings derived from the passive LFP sensing data.

### Network Activity

**Figure 8:**
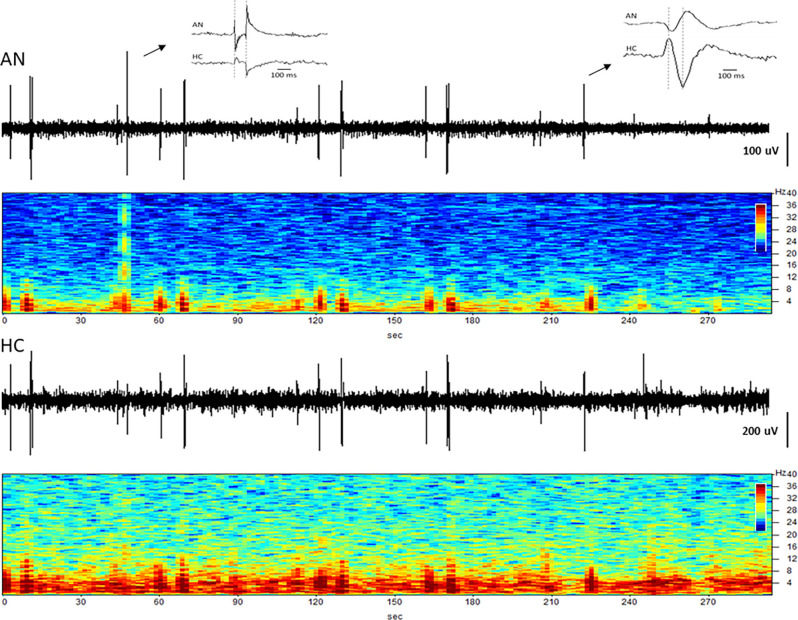
Coupling between the hippocampus and anterior nucleus. Time domain and spectrograms from simultaneously recorded signals from the AN (top) and HC (bottom) in subject C3 (Year 4). Spike activity originating in the HC is recorded 30–40 ms later in the AN (right inset), consistent with evoked potential (EP) latency measures. The left inset shows a K-complex recorded as a large signal in the AN, but a small signal with no latency difference in HC, suggesting a far-field recording at that site.

The simultaneous LFP recordings from the thalamus and hippocampus often showed coupled patterns of activity within this network, as described previously (Stypulkowski et al., 2013). Typically, during periods of high theta activity in the hippocampus, generally considered to be an “input” state, there was little coincident activity in the thalamus. In contrast, during periods of hippocampal sharp wave activity, an “output” state where information is passed to cortical and sub-cortical regions, it was common to observe parallel changes in thalamic firing. An interesting recording from subject C3 captured approximately 4.5 years post-implant is shown in Figure 8. This record was obtained at roughly 1 a.m. (lights out) and presumably reflects a sleep state. Large amplitude spikes can be seen in the hippocampus with co-incident spikes observed in the thalamic recording. At an increased resolution (right inset) it is apparent that the spikes in the hippocampus lead the activity in the AN by approximately 30–40 ms, suggesting that these events originate in the hippocampus and propagate *via* the fornix outflow to the thalamus. In contrast, early in this recording, there was a large amplitude event in the AN record (left inset) with only a small co-incident spike in the hippocampus. This thalamic event is consistent with the appearance of a K-complex, which is generated in cortical regions during specific sleep stages and propagates to the thalamus (Wennberg, 2010). The inset shows the timing of this spike event to be essentially simultaneous at the two recording sites, which, along with the small amplitude, suggests that it was likely a far-field recording of this event at the hippocampal electrode.

## Discussion

Closed loop stimulation is a promising approach for advancing DBS therapies, with the ultimate goal of improving efficacy and reducing side effects. The ability to deliver a successful aDBS therapy clinically will depend critically upon two factors: (1) identification of a dependable control signal that provides an indication of the patients’ symptomatic state; and (2) the ability of the implanted hardware to reliably and durably monitor this signal to inform the therapy control algorithm.

With respect to Parkinson’s disease, the magnitude of specific frequency bands of cortical and subcortical LFP signals appears to correlate reasonably well with the clinical state (Brown, 2006). Both therapeutic levels of medication (Silberstein et al., 2003) and DBS (Kühn et al., 2008) suppress excessive low-frequency LFP signals in parallel with improvement of bradykinesia and rigidity (Ray et al., 2008). In contrast, high-frequency LFP components associated with dyskinesias are increased by peak medication doses or over-stimulation (Swann et al., 2018a). Although most of these studies have been conducted over relatively short time periods, raising questions of long-term applicability, subcortical LFP signals have been demonstrated to be stable over many years when recorded at different intervals from externalized DBS leads (Abosch et al., 2012).

Our current results suggest that it should also be possible to record similarly stable LFP signals from sub-cortical or cortical sites, over long periods of time, with this fully implanted system. This sets up the key question on how this will translate to closed loop feature development. The first step in closed loop feature development lies in having a stable therapy and measurement system which this work supports. The next step in that process is understanding the stability of the biological signals clinically. Questions like, how do those biological signals change with disease progression for example. Initial reports of clinical experience with these implantable devices have provided encouraging near-term performance results (Quinn et al., 2015; Trager et al., 2016; Neumann et al., 2017). Long-term results with a responsive device to treat epilepsy using cortical signals also support the ability of fully implantable systems to record neural activity over multiple years (Geller et al., 2017). Although the multi-electrode single unit recording arrays used for brain-machine interfaces can provide extremely rich information content, their long-term stability has been a recurring issue in experimental and clinical applications (Barrese et al., 2013). Instead, DBS or surface macroelectrodes may provide more reliable long-term access to the appropriate neural control signals for mainstream clinical applications.

Lead and extension integrity is a key component to the long-term stability of neural recordings. In this work, four of the seven subjects exhibited multiple lead conductor breakages over the course of the investigation, which would be unexpected in a human clinical setting. Upon analysis of post-operative images, it was determined that the veterinary surgeon who implanted these systems had placed the connectors of the DBS extensions in the neck region of the animals due to limited space at the skull surgical site which contained the two burr hole caps (Figure 1). In human surgeries, the extension connector is always placed on the side of the head so that the extension, and not the lead body, is exposed to flexion due to head and neck motion. This is based on the early clinical experience just after DBS was first approved, where multiple lead fractures were observed when connectors were placed in the neck (Schwalb et al., 2001; Hariz, 2002). Moreover, a recent study in this same ovine animal model also reported a high incidence of DBS lead fractures found to be related to connector location (Lentz et al., 2015).

The materials and construction of the DBS extensions are by design, intended to be resistant to fatigue from repeated flexion cycles. The DBS leads, however, use different materials and construction and the lead conductors are more susceptible to mechanical fatigue. The placement of the lead-extension connection in the muscular neck region of these animals appeared to be the cause of the lead breakages that were experienced. Despite these failures, it was possible in most cases through programming changes to stimulation or recording configurations, to re-capture evoked responses or LFPs similar to those obtained with the original contact configurations.

We previously described the concept of local and remote modulation within this Papez circuit, as it related to the treatment of epilepsy, by using either direct hippocampal stimulation or anterior thalamic DBS, to influence hippocampal excitability (Stypulkowski et al., 2014) and recently extended those findings to a second cohort of subjects with leads in the hippocampus and fornix (Stypulkowski et al., 2017). In both cases, recording of LFP activity from the hippocampus provided insight into the effects of DBS at different network nodes on this target structure. Moreover, it was possible to demonstrate the use of these hippocampal LFPs as a control signal for closed-loop stimulation, delivered from either the local or remote stimulation sites. The results shown in Figure 3 provide encouraging data regarding the long-term stability of these types of recordings as well as the apparent stability of the DBS thresholds to induce both inhibitory and excitatory effects within this network. This demonstration of both recording and stimulation threshold stability over a period of several years supports the viability of these methods for clinical use. The approach of employing a remote recording electrode within the targeted therapy network, to monitor the effects induced by DBS at a second site, has recently been clinically investigated for both epilepsy (Van Gompel et al., 2015) and movement disorders (Shute et al., 2016; Swann et al., 2018a) using the implantable device described here.

New lead geometries with segmented electrodes are now available for some approved DBS therapies. These new directional lead designs allow for more detailed information to be collected using LFP sensing. An intraoperative study in Parkinson’s disease patients that measured monopolar LFP data from traditional cylindrical contacts in the Subthalamic Nucleus (STN) showed that LFP beta power correlated with the top-ranked electrodes for clinical efficacy (Aman et al., 2020). More recently, another study using externalized directional leads demonstrated that individual Parkinson’s patients had unique frequency spectrum patterns that varied across the lead, and that the contacts with the highest LFP amplitude matched the electrodes selected for therapy (Tinkhauser et al., 2017). These studies show the potential value of LFP sensing from DBS leads and the possibility of using it as a clinical tool. Our current study showed that LFP sensing on directional leads can be obtained from a fully internalized DBS system and that these signals can be obtained chronically. Encouragingly, the preliminary results from the directional stimulation experiments suggest that passive LFP sensing may be useful in the Papez network to identify electrodes that produce the greatest response to stimulation, consistent with the results reported in STN DBS.

The current study has clear limitations including the small sample size. However, the ability to record chronically for very long periods of time with repeated measures in the same subjects is a unique opportunity not afforded with acute or semi-chronic (percutaneous) experimental approaches. With the exception of the unanticipated, but explained lead breakages, the implanted hardware performed as expected over the course of the study. Electrode impedance measurements were taken over time using the implanted device. The implanted device delivers a constant current stimulus output and measures the resulting voltage output for that waveform using an analog to digital converter (ADC). Stimulation is delivered at 100 Hz and 80 μs. By knowing the delivered constant current and measured voltage, the implanted device calculates |Z|=|V/I|. A representative trend is shown in Figure 6. Electrode impedances followed typical patterns as reported by others (Sillay et al., 2010; Cheung et al., 2013) with generally stable impedances after a period of initial stabilization. Contacts that were used for stimulation typically exhibited lower, and more stable impedance than those used purely for passive recordings (e.g., by a factor of 2–3 over the long-term; Satzer et al., 2014). In addition, the input impedance of the recording amplifier is set by the high pass filter and in the Megaohm range (Stanslaski et al., 2012). Given this high input impedance, normal impedance variations do not attenuate the measures signals. Local field potential recordings were consistent over the course of the study, with no apparent systematic degradation of signal quality. Evoked responses and simultaneous recordings from the two target sites demonstrate the ability to measure and monitor the strength of network connectivity with this implanted system for periods of up to 5 years. As DBS therapies continue to evolve and expand into new indications, this potential to monitor the targeted neural network for acute and chronic effects of stimulation may become more important (Freestone et al., 2013). With respect to closed-loop DBS for current movement disorder therapies, the system appears to be a viable platform for long-term delivery of such a therapy. Initial studies using this system to test closed-loop DBS in human subjects have recently been reported (Swann et al., 2018b) and the first clinical investigations employing chronic closed-loop DBS are anticipated.

## Data Availability Statement

The raw data supporting the conclusions of this article will be made available by the authors upon request, without undue reservation.

## Ethics Statement

The animal study was reviewed and approved by Medtronic PLC ethics committee.

## Author Contributions

SS, MC, and PS: manuscript preparation, data collection and analysis. JG: data collection and analysis. RR: manuscript review and edit. All authors contributed to the article and approved the submitted version.

## Conflict of Interest

All of the authors are employees of Medtronic PLC.

## Publisher’s Note

All claims expressed in this article are solely those of the authors and do not necessarily represent those of their affiliated organizations, or those of the publisher, the editors and the reviewers. Any product that may be evaluated in this article, or claim that may be made by its manufacturer, is not guaranteed or endorsed by the publisher.
